# Defect Detection Method of Carbon Fiber Sucker Rod Based on Multi-Sensor Information Fusion and DBN Model

**DOI:** 10.3390/s22145189

**Published:** 2022-07-11

**Authors:** Chenquan Hua, Siwei Chen, Guoyan Xu, Yang Chen

**Affiliations:** College of Control Science and Engineering, China University of Petroleum (East China), Qingdao 266580, China; z20050031@s.upc.edu.cn (S.C.); z20050004@s.upc.edu.cn (G.X.); chenyang@hz.shandong.cn (Y.C.)

**Keywords:** carbon fiber sucker rod, multi-sensor information fusion, deep belief network, image identification, defect identification

## Abstract

Because of its unique characteristics of small specific gravity, high strength, and corrosion resistance, the carbon fiber sucker rod has been widely used in petroleum production. However, there is still a lack of corresponding online testing methods to detect its integrity during the process of manufacturing. Ultrasonic nondestructive testing has become one of the most accepted methods for inspection of homogeneous and fixed-thickness composites, or layered and fixed-interface-shape composites, but a carbon fiber sucker rod with multi-layered structures and irregular interlayer interfaces increases the difficulty of testing. In this paper, a novel defect detection method based on multi-sensor information fusion and a deep belief network (DBN) model was proposed to identify online its defects. A water-immersed ultrasonic array with 32 ultrasonic probes was designed to realize the online and full-coverage scanning of carbon fiber rods in radial and axial positions. Then, a multi-sensor information fusion method was proposed to integrate amplitudes and times-of-flight of the received ultrasonic pulse-echo signals with the spatial angle information of each probe into defect images with obvious defects including small cracks, transverse cracks, holes, and chapped cracks. Three geometric features and two texture features from the defect images characterizing the four types of defects were extracted. Finally, a DBN-based defect identification model was constructed and trained to identify the four types of defects of the carbon fiber rods. The testing results showed that the defect identification accuracy of the proposed method was 95.11%.

## 1. Introduction

**Figure 1:**
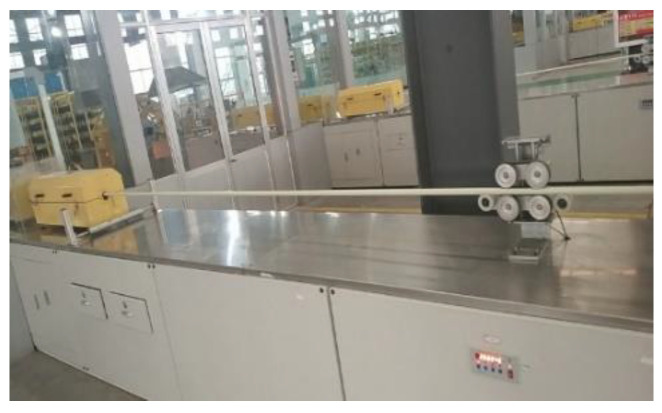
Manufacturing process of carbon fiber sucker rod.

The carbon fiber sucker rod is a typical composite material, which has the characteristics of small specific gravity and high strength and corrosion resistance, and it has been widely used in oil production sites [1,2], as shown in Figure 1. Some defects within the carbon fiber sucker rod may arise during the manufacturing process, leading to a significant decline in its performance and even serious accidents during the process of application, so the online real-time defect detection of the carbon fiber rod during the process of manufacturing is an urgent problem to be solved [3].

**Figure 2:**
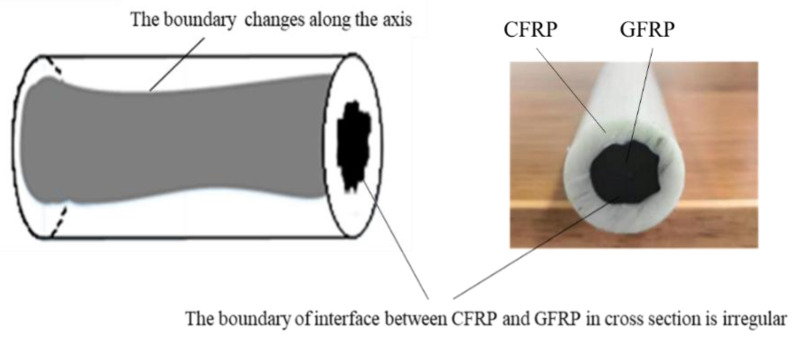
Carbon fiber sucker rod.

The carbon fiber sucker rod is a typical composite material with a carbon fiber pultruded multi-layer structure, as shown in Figure 2. It is a hybrid fiber-reinforced-polymers (FRPs) structure with a 22 mm diameter that combines the advantages of a carbon-fiber-reinforced polymer (CFRP) core with a diameter of about 16 mm and longitudinal sound velocity of about 2800 m/s, and a glass-fiber-reinforced polymer (GFRP) with a longitudinal sound velocity of about 3300 m/s. The boundary of interface between the CFRP and GFRP in cross-section is irregular, and the boundary changes along the axis. Due to the irregular interfaces between the CFRP and GFRP, defect inspection of the carbon fiber sucker rod is very difficult.

Some Nondestructive Testing (NDT) techniques have been developed for composite diagnostic purposes [4,5,6]. Because Ultrasonic Nondestructive Testing (UNDT) has the advantages of high detection accuracy, convenient use, simple operation, and wide application range [7], it has become one of the most accepted methods for composite inspection. There are many studies on the application of this method to composite inspection. Andrzej [8] tested three composite structures made of glass-fiber-reinforced plastic, a hybrid composite with a core made of the same material, and face sheets made of aluminum alloy and a carbon-fiber-reinforced plastic structure by using various techniques including PZT sensing, ultrasonic, thermography, and vibration-based inspection, but the testing is not suitable for online testing of the carbon fiber sucker rod with irregular interface multilayer composites. Cerniglia [9] proposed a laser ultrasonic technique for the in-line inspection of laser powder deposition components. Asokkumar [10] compared different air-coupled ultrasonic testing methods to characterize impact-type defects in a pultruded quasi-isotropic GFRP composite plate. However, this method is not suitable for the carbon fiber rod with the irregular interlayer interface. Zeng [11] presented a hybrid system for detecting the microstructure and defects in the braided CFRP that combines the advantages of laser-ultrasound and air-coupled ultrasonic testing. Caminero [12] used phased-array ultrasonic testing for the inspection of carbon-fiber-reinforced epoxy (CFRP) composite laminates with barely visible impact damage. Castellano [13] performed ultrasonic goniometric immersion tests in order to characterize mechanically the substrate and the new material obtained by the additive manufactured process. Combined with the CT imaging principle, Zhou [14] developed an imaging method based on probabilistic damage for the Lamb wave. However, this method has strict requirements on the thickness of the material to be examined and is not applicable to carbon fiber rods with an irregular interlayer interface. To improve the ultrasonic testing capability for additively manufactured materials, Song [15] proposed the time-dependent threshold to distinguish effectively the flaw echoes from the background of structural noise. Meng [16] proposed a deep learning model based on the convolutional neural network to classify carbon fiber polymer defects, and the wavelet packet method to extract features. Nguyen [17] divided the original casting visual image into nine regions in equal proportions, and then input it into the CNN to detect inclusions, pores, and cracks on the surface of the casting. Khumaidi [18] collected ultrasonic inspection images of defects in weldments as input, and used convolutional neural networks to achieve feature extraction and classification.

**Figure 3:**
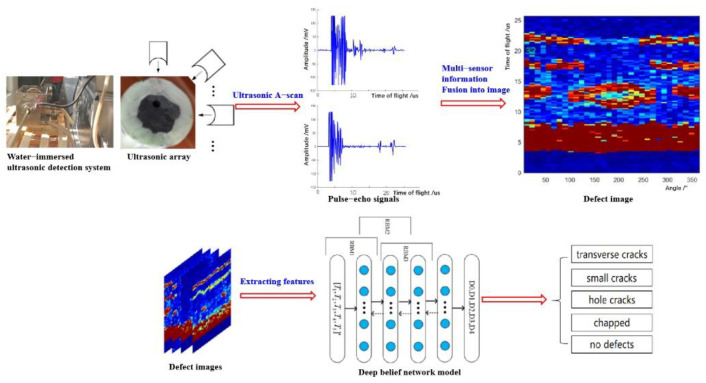
Flow chart of defect detection and identification.

According to the characteristics of irregular interfaces in the sucker rod and the requirements for follow-up online detection, in this paper, a novel alternative defect detection method based on the multi-sensor information fusion and deep belief network model was proposed to identify online its defects, as shown in Figure 3. A water-immersed ultrasonic array with 32 ultrasonic probes was designed to realize the online and full-coverage scanning of carbon fiber rods in radial and axial positions. Then, a multi-sensor information fusion method was proposed to integrate the amplitude and time-of-flight of received ultrasonic pulse-echo signals with the spatial angle information of each probe into an ultrasonic defect image with obvious defects including small cracks, transverse cracks, holes, and chapped cracks. A feature extraction method combining geometric features with texture features was proposed to extract three geometric features and two texture features characterizing the four types of defects from defect images. Finally, a DBN-based defect identification model was constructed and trained to identify the four types of defects of the carbon fiber rods.

## 2. Experimental Setup and Testing

### 2.1. Water-Immersed Ultrasonic Detection System

**Figure 4:**
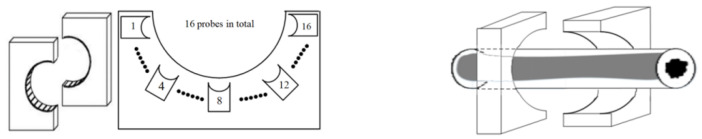
Schematic diagram of ultrasonic array detection.

According to the characteristics of irregular interfaces in the sucker rod and the requirements for online inspection, a water-immersed ultrasonic detection system with 32 probes was specially designed to perform ultrasonic inspection, as shown in Figure 4. Immersion testing provides testing flexibility as the sucker rod can move axially freely underwater with the fabrication process and introduce a sound wave at different angles without contacting the probes. The ultrasonic array includes 32 probes, and each probe is an ultrasound focus probe with a center frequency of 2 MHz and focal column diameter of 2.2 mm, which ensures 360-degree full-coverage inspection of the sucker rod. The 32 probes were cyclically scanned for inspection of carbon fiber rods in different radial angles with a 20 ms/s sampling rate and a 16 ms cycle time.

### 2.2. Experimental Testing Based on Ultrasonic Array

**Figure 5:**
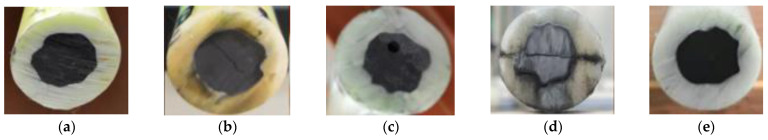
Carbon fiber sucker rod: (**a**) small crack defect, (**b**) transverse crack defect, (**c**) hole defect, (**d**) chapped defect, and (**e**) with no defect.

Four representative defects of the carbon fiber sucker rod including small cracks, transverse cracks, holes, and chapped cracks were selected for ultrasonic testing in this experiment, as shown in Figure 5. The full coverage scan of the sucker rods in the cross-section was performed, ultrasonic waves to encounter the interface with different acoustic impedance materials were reflected, and the corresponding ultrasonic reflection echoes were received by the same probes.

**Figure 6:**
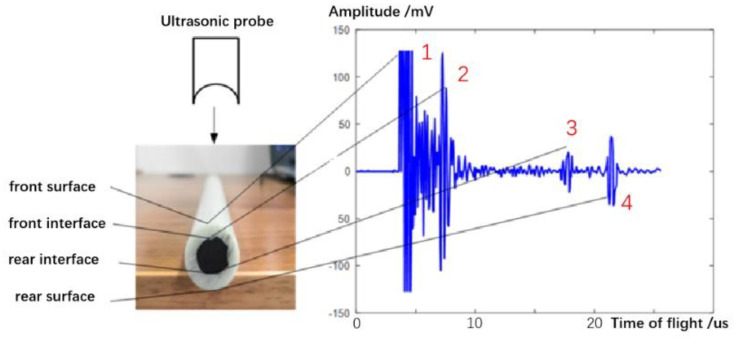
Ultrasonic pulse-echo signal of carbon fiber rod with no defect.

The ultrasonic pulse-echo signal uses the abscissa to represent the time-of-flight, and the ordinate to represent the amplitude of the pulse-echo signal. Therefore, the internal information of the material to be measured can be judged by the number and position of reflected echoes. Each pulse peak of the pulse-echo signal represents the reflected echoes from the interface between two materials with a significant difference of acoustic impedance. When a single probe detects the intact carbon fiber rod, the ultrasonic reflection signal is obtained, as shown in Figure 6. When the ultrasonic wave emitted by the probe enters the carbon fiber rod, it generates the 1# reflected echo at its front surface; when the ultrasonic wave passes through the front interface of the carbon fiber rod, it generates the 2# reflected echo due to the change in impedance; when the ultrasonic wave passes through the rear interface of the carbon fiber rod, it generates the 3# reflected echo; when the ultrasonic wave propagates to the rear surface of the carbon fiber rod, it generates the 4# reflected echo. As a result, for the intact carbon fiber rod, four reflected echoes corresponding to each interface of the carbon fiber rod are received.

**Figure 7:**
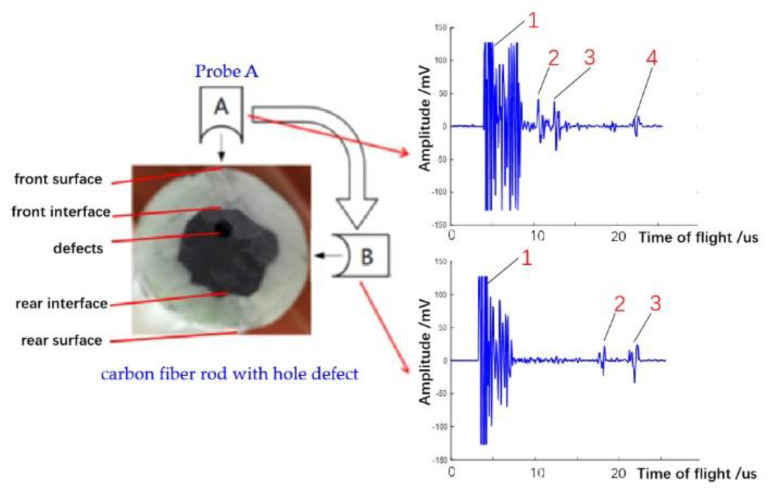
Ultrasonic reflection signal of carbon fiber rod with hole defect.

In view of the characteristics of irregular interfaces in the sucker rod, an ultrasonic array detection system containing 32 probes was designed to scan different angles from the cross-section of the sucker rod, and the corresponding ultrasonic reflection waves from different angles carry different defect information because the propagation direction of ultrasonic waves is different. We take the hole defect as an example, as shown in Figure 7. The reflected echoes of the front surface and the front interface of the carbon fiber rod mix together to form a long-lasting reflected wave, that is, the 1# waveform. Then, the ultrasonic waves propagate to the hole defect to form the 2# reflected echo, that is, the defect echo. Then, the ultrasonic waves reach the back interface of the carbon fiber rod to form the 3# reflected echo. Finally, the ultrasonic waves propagate to the back surface of the carbon fiber rod to form the 4# reflected echo. Compared with the intact carbon fiber rod, the reflected echoes of the carbon fiber rod with the hole defect have one more defect echo.

The ultrasonic wave emitted by probe A vertically passes through the defect area, and the ultrasonic reflected wave contains the defect echo. However, the ultrasonic wave emitted by probe B does not pass through the defect area, and the obtained ultrasonic reflection signal does not contain the defect echo, as shown in Figure 6. Moreover, because the interface between the CFRP and GFRP of the carbon fiber rod is irregular, the ultrasonic echoes of the interface obtained by different probes emitting ultrasonic waves from different positions are also different.

Experiments for carbon fiber sucker rods with no defect and four typical defects were carried out, and 750 samples were obtained, including 150 samples for each defect type and no defect. Each sample included 32 ultrasonic pulse-echo signals.

## 3. Multi-Sensor Information Fusion into Defect Images

**Figure 8:**
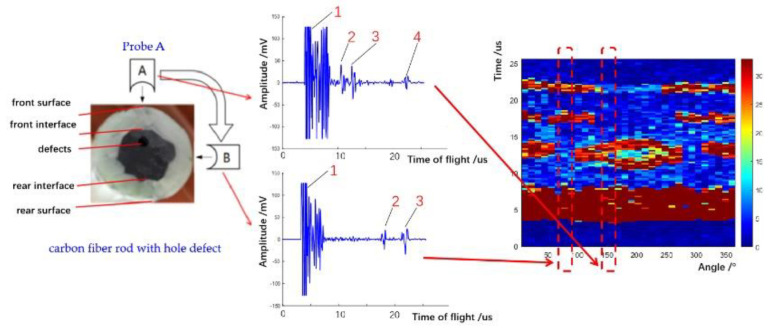
Multi-information fusion into an image.

A multi-sensor information fusion method was proposed to integrate the amplitude and time-of-flight of received ultrasonic pulse-echo signals with the spatial angle information of each probe into defect images with obvious defects including small cracks, transverse cracks, holes, and chapped cracks. Taking the hole defect as an example, as shown in Figure 8, the angles, amplitudes, and time-of-flight of the received ultrasonic pulse-echo signals can be visually reflected on one image. The abscissa represents the angle corresponding to the ultrasonic probe, the ordinate represents the time-of-flight, and the color in the image represents the amplitude of signals.

**Figure 9:**
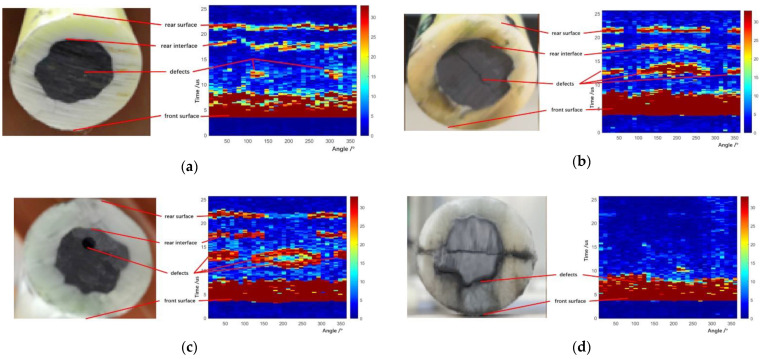
Ultrasonic images of sucker rods with different defects: (**a**) small crack defect, (**b**) transverse crack defect, (**c**) hole defect, and (**d**) chapped defect.

The cross-sectional defect image can be obtained when the ultrasonic probes complete the radial scanning of the carbon fiber rod. With the movement of the carbon fiber rod, the detections of the whole carbon fiber rod were completed, which meets the requirements of online detection of the carbon fiber rod. In addition, the received pulse-echo signals with different defect information were mapped into different defect images, in which the ultrasonic probe angles, the amplitude, and the ultrasonic time-of-flight of the received pulse-echo signals were fused. Then, many image recognition methods can be used to identify the defects. Ultrasonic images of sucker rods with four different defects are shown in Figure 9.

## 4. Feature Extraction of Defect Images

### 4.1. An Image Enhancement Method Combining Bilateral Filtering and Laplace Operator

**Figure 10:**
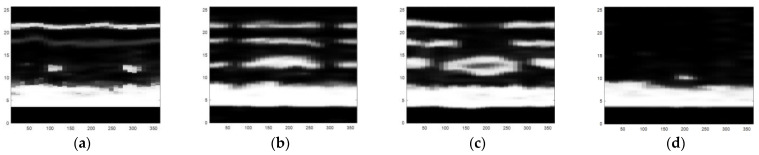
The defect images processed by bilateral filtering: (**a**) small crack, (**b**) transverse crack, (**c**) hole defect, and (**d**) chapped defect.

**Figure 11:**
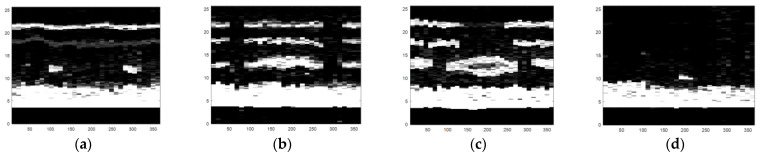
Grayscale images of four types of defects: (**a**) small crack, (**b**) transverse crack, (**c**) hole defect, and (**d**) chapped defect.

**Figure 12:**
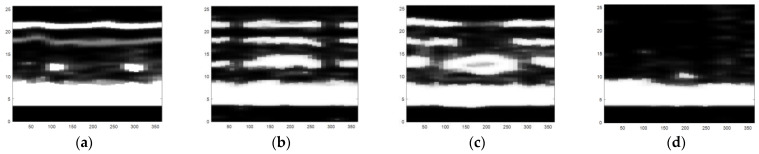
The defect images processed by the combination of Laplacian operator and bilateral filtering: (**a**) small crack, (**b**) transverse crack, (**c**) hole defect, and (**d**) chapped defect.

The obtained ultrasonic defect images from above are a more visual expression for different defects of carbon fiber sucker rods, and then an image identification method based on the deep belief network model can be used to identify different defects of carbon fiber rods. After gray processing [19], an image enhancement method combining bilateral filtering and the Laplace operator was proposed, as shown in Figure 10, Figure 11 and Figure 12.

**Figure 13:**
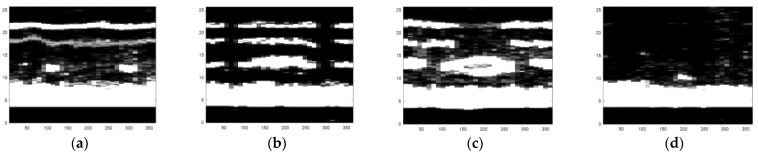
The defect images processed by the Laplacian operator: (**a**) small crack, (**b**) transverse crack, (**c**) hole defect, and (**d**) chapped defect.

A significant feature of the image edge is the mutation of pixel value. As the derivative can represent a sharp change in pixel gray value on an image edge, image sharpening can be realized by using the differential operator. Commonly used differential operators include the first-order differential operator Sobel operator and second-order differential operator Laplace operator. As the Sobel operator has less response to isolated points in the image than the Laplace operator does [20], the Laplace operator is adopted to process the grayscale image by Laplace operator sharpening, as shown in Figure 13. Compared with Figure 11, the pixel brightness of the defect area and the interface area changes significantly, but the existing noise in the image is enhanced and can be misjudged as the defect area. We focus on the image surface, interface region, and defect region, which need to retain the edge information during denoising. In view of the fact that bilateral filtering can retain image edge details during filtering, bilateral filtering is used to suppress image noise and compensate for the problem of excessive enhancement of noise area during image enhancement by the Laplace operator [21].

As can be seen from Figure 10, the noise in the image is significantly reduced while the defects, surfaces, and edges of the interface region of the image are retained. Therefore, in order to avoid the misjudgment of defect area caused by using the Laplace operator alone, it is necessary to combine bilateral filtering with the Laplace operator, to preserve the boundary information and defect edge information in the image and reduce noise. The image enhancement effect of the combination of bilateral filtering and Laplace operator is shown in Figure 12. The image processing in Figure 12 sharpens the defect areas and boundaries to help to remove invalid areas and extract features in Section 4.2.

### 4.2. Defect Image Segmentation and Invalid Area Elimination

**Figure 14:**
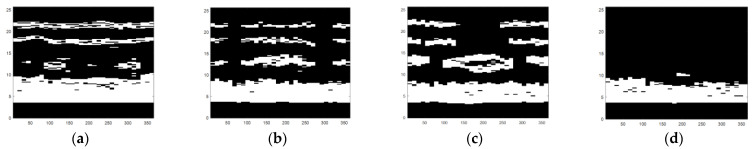
The defect images processed by the three-threshold maximum inter-class variance method: (**a**) small crack, (**b**) transverse crack, (**c**) hole defect, and (**d**) chapped defect.

The image enhancement method above may sharpen the defect regions and boundaries. However, in order to better extract the defect features, we also need to remove the irrelevant areas. Aiming at the feature that the pixel value of the rear interface of the carbon fiber rod image is too small, the three-threshold maximum interclass variance method is used for image segmentation. As shown in Figure 14, four types of defect images obtained the effective segmentation, especially after the interface region; they will no longer be mistaken for the background region, because the three most between-cluster variance threshold method uses different threshold segmentations for different areas of the image. However, there are still two invalid regions in the image: impurity region and to-be-filled region. In order to facilitate feature extraction, these two types of invalid regions need to be removed.

**Figure 15:**
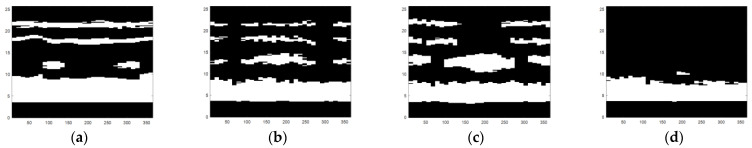
The defect images after removing the invalid area: (**a**) small crack, (**b**) transverse crack, (**c**) hole defect, and (**d**) chapped defect.

As can be seen from Figure 15, redundant invalid areas are effectively removed and only the front surface, back interface, back surface, and defect area of the carbon fiber rod are retained. All boundary information and defect information in the image can be well identified, providing better conditions for subsequent feature extraction.

### 4.3. A Feature Extraction Method Combining Geometric Features with Texture Features

A feature extraction method combining geometric and texture features for sucker rod defects was used. Three geometric features and two texture features were well-chosen and extracted to represent typical defect features.

#### 4.3.1. Extraction of Geometric Features of Defect Images

According to the characteristics of carbon fiber rods, area sum, maximum width sum, and length sum can effectively improve the accuracy of defect classification and identification.

For the four types of defects, there are different connected domains that represent the different echo-interfaces: front surface, rear surface, rear interface, or defect interface. The area S of the connected domain is expressed by the number of pixels contained in the connected domain. *X* is the abscissa of the center of mass of the connected domain, which represents the radial angle surrounded by the connected domain in °. *Y* is the ordinate of the center of mass of the connected domain, which represents the time of ultrasonic echo in μs.

The width of the connected domain represents the time it takes for the ultrasonic echo to appear and disappear in μs. The length of the connected domain is the abscissa length of the connected domain of the image, which represents the radial angle surrounded by the connected domain in °. For different types of defect images, the widths and lengths of the different echo-interfaces have different characteristics.

The length of the connected domain is the length of the connected domain on the abscissa of the image. The concepts of length and sum of length are introduced to indicate how many probes a defect can be detected by. For different types of defect images, the length and length of defect regions have different characteristics.

The geometric characteristic information of four typical defects is shown in Table 1, Table 2, Table 3 and Table 4. It can be seen that the three geometric features, area, length, and maximum width, can effectively describe the defect area characteristics of small cracks, transverse cracks, and holes, but cannot effectively characterize the characteristics of crack defects.

#### 4.3.2. Extraction of Texture Features of Defect Images

In order to describe the features of chap defects effectively, texture features are introduced in this paper. Texture features are widely used in the description of image information to describe image laws from a macroscopic perspective. The gray co-occurrence matrix is a common texture feature analysis method used to reflect the comprehensive information of image gray level [22]. In this paper, the four statistics of energy, entropy, correlation, and contrast constructed by the gray co-occurrence matrix were used to describe texture features. When constructing the gray level co-occurrence matrix, co-occurrence matrices in the four directions of 0 degrees, 45 degrees, 90 degrees, and 135 degrees were selected; the characteristic quantities of the gray level co-occurrence matrix in these four directions were obtained; their mean values were finally obtained [23].

As shown in Table 5, the difference in energy mean, entropy mean, contrast mean, and correlation mean value of D1, D2, and D3 is small, while the texture characteristics of D4 and the other three types of defects are quite different, because the number of boundaries between D4 and other defect images has the same big difference. There are few interface areas that can detect D4, so its contrast is smallest; the correlation reflects the local similarity of the image, D4 has fewer internal boundaries, and the local similarity is high, so its correlation average is largest. Therefore, the two texture features with maximum difference, contrast mean and correlation mean, can effectively characterize the characteristics of D4.

As shown in Table 6, the three geometric features can effectively distinguish small cracks, transverse cracks, and hole cracks, and the two texture features can effectively distinguish chap defects from other defects. Therefore, the features including the sum of defect lengths, the sum of defect areas, the maximum defect width, the contrast mean, and the correlation mean can characterize the characteristics of different defects, and can then be used to identify the different defects of carbon fiber sucker rods. The feature vector *T* for defect identification can be expressed:

(1) $$T={\left[T_{1},T_{2},T_{3},T_{4},T_{5}\right]}^{\prime }$$

where the features *T*_1_, *T*_2_, *T*_3_, *T*_4_, and *T*_5_ represent, respectively, the sum of defect lengths, the sum of defect areas, the maximum defect width, the average contrast, and the correlation mean. The five features of some defect samples for carbon fiber sucker rods are shown in Table 6.

## 5. DBN-Based Defect Identification Model

There are many networks that can be selected for deep learning application in structural testing, such as the Deep belief network (DBN) and convolutional neural network (CNN) [24]. Because the DBN reconstructs original data through unsupervised training, unlabeled samples can be used for model training, so it is suitable for processing data with no significant correlation of adjacent features [25].

**Figure 16:**
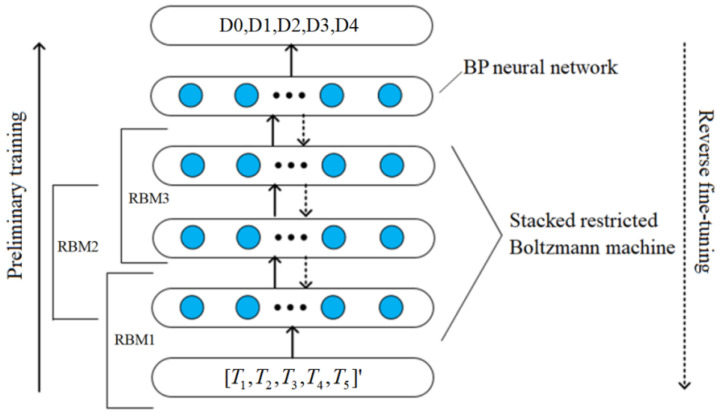
Defect identification model based on deep belief network.

A DBN-based defect identification model was constructed to realize the defect identification of the carbon fiber sucker rod, as shown in Figure 16. The feature vector T was set as inputs of the model with five input neuron nodes, while four different defects and no defect were used as outputs of the model with five output neuron nodes. The Sigmoid function was selected as the activation function [26]. According to experience, the learning rate of each RBM in the pre-training process is generally set at 0.1, while the learning rate of the BP algorithm is generally set at 0.01 [27]. After experimental testing, the number of hidden layers was selected as 3, and the number of neuron nodes in each hidden layer was 35. DBN construction and training were divided into three processes: parameter setting, pre-training, and fine-tuning. The final defect identification model was obtained by unsupervised pre-training and the supervised backpropagation algorithm. Datasets with 150 samples were collected for each of the four types of defects and no defect. A percentage of 70 percent of them were selected as the training set and 30 percent as the test set.

The DBN-based model was trained for the trained dataset and tested for the testing dataset. The testing results show that the defect identification accuracy of the proposed method is 95.11%, as shown in Table 7. For chapped defects, the texture features are obvious, so the identification rate is high. However, due to bilateral filtering in the process of defect image processing, some useful information in the ultrasonic B-scan image is removed. Especially for hole defects, due to the random location, random size, and irregular edge of holes, the defect area changes greatly. When extracting geometric features, it is close to small cracks and transverse cracks that affect the accuracy of identification.

## 6. Conclusions

Due to their light weight, high strength, and corrosion resistance, carbon fiber sucker rods are widely used in oil production. However, there is still a lack of effective online testing methods to detect its integrity during the process of manufacturing. In this paper, a novel DBN-based defect detection method based on ultrasonic multi-sensor information fusion was proposed to identify online its defects. Based on the results of the above data processing and analysis, the following conclusions can be made:

(1) According to the characteristics of irregular interlayer interfaces in carbon fiber sucker rods and the requirements for online inspection, a water-immersed ultrasonic detection system with 32 probes was specially designed to perform ultrasonic inspections in different radial and axial positions, in which the sucker rod can move freely underwater with the fabrication process and introduce a sound wave at any desired angle without contacting the probes.
(2) A multi-sensor information fusion method was proposed to integrate amplitudes and times-of-flight of the received ultrasonic pulse-echo signals with the spatial angle information of each probe into defect images with obvious defects including small cracks, transverse cracks, holes, and chapped cracks. From this, many common image recognition methods can be used to identify the defect types from the defect images.
(3) A feature extraction method combining geometric features with texture features was proposed to extract three geometric features and two texture features characterizing the four types of defects from the defect images. Then, the features can be used to construct the model identifying the defects.
(4) A DBN-based defect identification model was constructed and trained to identify the four types of defects of the carbon fiber rods. The testing results show that the defect identification accuracy of the proposed method is 95.11%.

However, the dataset of the four typical defects from the experiment is not enough, which may lead to a decrease in accuracy due to the weak network generalization ability. In future trials, if supervised learning methods are adopted, such as using the convolutional neural network to directly identify b-scan images obtained, the hole defects by which errors are easy to identify can be labeled in advance to further improve the recognition rate.

## Figures and Tables

**Table 1:** Geometric feature information of selected small crack defect image.

	X	Y	S	Length	Width	Maximum Width	Sum of Length	Sum of Area
	(°)	(μs)	(No.)	(°)	(μs)	(μs)	(°)	(No.)
Front surface	184.61	6.03	740	360.00	5.40	5.40	360.00	740
Rear surface	178.99	21.41	139	360.00	1.60	1.60	360.00	139
Rear interface	188.10	17.82	116	360.00	2.25	2.25	360.00	116
Defect	111.26	12.17	35	56.25	1.85	1.85	123.75	73
	296.78	12.08	38	67.50	1.80			

**Table 2:** Geometric feature information of selected transverse crack defect image.

	X	Y	S	Length	Width	Maximum Width	Sum of Length	Sum of Area
	(°)	(μs)	(No.)	(°)	(μs)	(μs)	(°)	(No.)
Front surface	183.26	6.17	759	360.00	5.60	5.60	360.00	759
Rear surface	15.96	21.48	12	22.50	1.45	1.45	225.00	74
	168.53	21.60	46	135.00	1.40			
	350.21	20.95	16	33.75	1.20			
Rear interface	11.36	18.30	6	11.25	1.25	2.20	146.25	66
	184.16	18.02	51	112.50	2.20			
	353.70	18.14	9	22.50	1.05			
Defect	25.76	12.91	17	45.00	1.20	2.60	281.25	195
	184.95	13.42	158	191.25	2.80			
	344.59	12.76	20	45.00	1.45			

**Table 3:** Geometric feature information of selected hole defect image.

	X	Y	S	Length	Width	Maximum Width	Sum of Length	Sum of Area
	(°)	(μs)	(No.)	(°)	(μs)	(μs)	(°)	(No.)
Front surface	184.16	5.92	717	360.00	5.65	5.65	360.00	717
Rear surface	68.85	21.86	62	135.00	2.00	2.00	258.75	124
	301.95	21.51	62	123.75	1.85			
Rear interface	21.83	17.82	17	33.75	1.60	2.00	213.75	111
	90.79	17.64	44	78.75	2.00			
	316.35	17.56	50	101.25	1.85			
Defect	32.06	12.35	53	56.25	2.60	4.45	292.50	344
	191.36	11.75	245	180.00	4.45			
	339.75	12.21	46	56.25	2.40			

**Table 4:** Geometric feature information of selected chapped defect image.

	X	Y	S	Length	Width	Maximum Width	Sum of Length	Sum of Area
	(°)	(μs)	(No.)	(°)	(μs)	(μs)	(°)	(No.)
Front surface	177.41	6.23	735	360.00	6.05	6.05	360.00	735
Defect	192.15	12.36	49	67.50	2.20	2.20	67.50	49

**Table 5:** Texture feature information of selected defect image.

Defect Type	Energy Mean	Entropy Mean	Contrast Mean	Correlation Mean
D1	0.5622	0.7911	0.2511	1.1007
D2	0.5501	0.8034	0.2470	1.0742
D3	0.4992	0.8791	0.2925	0.9593
D4	0.6628	0.6068	0.1081	1.4684

D0, D1, D2, D3, and D4 represent, respectively, small crack defects, transverse crack defects, hole defects, chapped defects, and no defects.

**Table 6:** Features of some defect samples.

	Sum of Length	Sum of Area	Maximum Width	Contrast Mean	Correlation Mean
	(°)	(No.)	(μs)		
D1	101.25	68	2.43	0.2389	1.063
	78.75	39	2.00	0.2511	1.101
	90.00	62	2.25	0.2493	1.057
	112.50	89	2.60	0.2352	1.055
	168.75	124	2.45	0.2340	1.032
D2	281.25	195	2.80	0.2470	1.074
	202.50	116	1.85	0.2084	1.085
	213.75	183	3.00	0.2118	1.081
	292.50	248	3.05	0.2214	1.062
	247.50	181	2.40	0.2234	1.093
D3	292.50	344	4.45	0.2925	0.959
	326.25	371	4.60	0.1986	1.086
	247.50	195	3.15	0.2088	1.098
	236.25	225	3.85	0.2249	1.103
	360.00	446	4.00	0.1623	1.111
D4	56.25	49	2.20	0.1081	1.468
	112.50	47	1.95	0.0805	1.689
	135.00	87	2.60	0.1065	1.446
	146.25	63	1.75	0.0810	1.581
	112.50	61	2.13	0.0940	1.546

**Table 7:** Comparison of defect classification accuracy.

	The Training Set		The Test Set	
	Wrong Number	Accuracy (%)	Wrong Number	Accuracy (%)
D0	0	100.00	0	100.00
D1	2	95.23	1	97.78
D2	5	92.38	3	93.33
D3	11	89.52	7	84.44
D4	0	100	0	100.00
Total number of errors	18		11	
The total accuracy (%)	96.57		95.11	

## Data Availability

Data is contained within the article.
